# Lack of association between *miR-218* rs11134527 A>G and Kawasaki disease susceptibility

**DOI:** 10.1042/BSR20180367

**Published:** 2018-05-22

**Authors:** Lei Pi, Lanyan Fu, Yufen Xu, Di Che, Qiulian Deng, Xijing Huang, Meiai Li, Li Zhang, Ping Huang, Xiaoqiong Gu

**Affiliations:** 1 Department of Clinical Biological Resource Bank, Guangzhou Institute of Pediatrics, Guangzhou Women and Children’s Medical Center, Guangzhou Medical University, Guangzhou 510623, Guangdong, China; 2 Department of Clinical Lab, Guangzhou Institute of Pediatrics, Guangzhou Women and Children’s Medical Center, Guangzhou Medical University, Guangzhou 510623, Guangdong, China; 3 Department of Cardiology, Guangzhou Women and Children’s Hospital, Guangzhou Medical University, Guangzhou 510623, Guangdong, China

**Keywords:** Kawasaki disease, miR-218, polymorphism, susceptibility

## Abstract

Kawasaki disease (KD) is a type of disease that includes the development of a fever that lasts at least 5 days and involves the clinical manifestation of multicellular vasculitis. KD has become one of the most common pediatric cardiovascular diseases. Previous studies have reported that *miR-218* rs11134527 A>G is associated with susceptibility to various cancer risks. However, there is a lack of evidence regarding the relationship between this polymorphism and KD risk. The present study explored the correlation between the *miR-218* rs11134527 A>G polymorphism and the risk of KD. We recruited 532 patients with KD and 623 controls to genotype the *miR-218* rs11134527 A>G polymorphism with a TaqMan allelic discrimination assay. Our results illustrated that the *miR-218* rs11134527 A>G polymorphism was not associated with KD risk. In an analysis stratified by age, sex, and coronary artery lesions, we found only that the risk of KD was significantly decreased for children older than 5 years (GG vs. AA/AG: adjusted OR = 0.26, 95% CI = 0.07–0.94, *P*=0.041). The present study demonstrated that the *miR-218* rs1113452 A>G polymorphism may have an age-related relationship with KD susceptibility that has not previously been revealed.

## Introduction

Kawasaki disease (KD) is an acute, systemic inflammatory vasculitis disease that is also known as lymphatic mucosa syndrome and usually affects infants and young children [1,2]. The clinical features of the disease mainly manifest as a fever lasting 5 days or longer. Patients should be given adequate treatment with intravenous immunoglobulin (IVIG) in the early stage. Coronary artery lesions (CALs) develop in more than 20–25% of untreated patients, some of whom even develop coronary artery aneurysms [3], which are the main cause of death in KD patients. After treatment, the complication rate decreases to 5%. Thus far, the etiology of KD remains unclear. Epidemiological studies have demonstrated that the incidence rates of KD are increasing year by year in the areas of Japan, South Korea, and Taiwan [4]. Most previous studies have focused on the associations of KD susceptibility with single nucleotide polymorphisms (SNPs), such as SNPs of *ITPKC, GRIN3A, ITPR3, ADAM17, CASP3, TARC/CCL17* etc. [5–9]. However, the relationships between microRNA polymorphisms and KD susceptibility have not been reported.

MicroRNAs (miRNAs) are a class of endogenous, small RNAs of approximately 18–25 nucleotides. One miRNA can have multiple target genes, and several miRNAs can also regulate the same gene. miRNAs exist in many forms, including pri-miRNAs, pre-miRNAs, and mature miRNAs. SNPs are spread throughout the human genome and play a main role in DNA mutations that are functional or regulate various biological processes and therefore can contribute to the development or prevention of diseases [10,11]. Numerous studies have revealed the relationship between the *miR-218* polymorphism and cancer risk in the Chinese population. Gao et al. [12] reported a meta-analysis about cancer in the Chinese population, and their results revealed that the *miR-218* rs11134527 A>G polymorphism is linked to a remarkable reduction in cancer risk in GG genotype people compared with AA and AG genotype people. Other studies have demonstrated that the *miR-218* rs11134527 A>G polymorphism is related to the susceptibility to cervical cancer, esophageal squamous cell carcinoma, breast cancer, and neuroblastoma [13–16]. Several studies have demonstrated the relationship between *mir-218* and cardiovascular disease. For example, *miR-218* has been reported to be associated with pathogenesis of myocardial infarction [17]. *miR-218* also regulates heart tube formation in zebrafish by targeting Robo family proteins [18]. Chiavacci et al. [19] demonstrated that *miR-218* mediates Tbx5 overexpression during zebrafish heart development. However, the association of the *miR-218* genetic polymorphism with KD is unknown. In the present study, we focused on whether the *miR-218* rs11134527 A>G polymorphism was related to the risk of KD.

## Materials and methods

### Study population

The subjects included 532 patients with KD and 623 controls that were collected from January 2012 to January 2017 in the Guangzhou Women and Children Medical Center in China. The KD patients were diagnosed mainly based on the Japanese Kawasaki Disease Research Association’s comprehensive clinical diagnostic criteria [20]. The KD patients attended our hospital as outpatients with follow-ups and inpatients, and the healthy controls were children who came to our hospital for health examinations within the same time period and had no fever or other diseases. The present study was approved by the Guangzhou Women and Children Medical Center Ethics Committee, and the children and their families provided written informed consent.

### DNA extraction and genotyping

Samples of 200 µl of anticoagulant-containing blood were collected according to the instructions of the Genomic DNA Extraction Kit. The specific procedures can be found in the literature [21–23]. The centrifuge tube was collected and quantified using a nucleic acid quantifier, which was eventually stored at −80°C. The *miR-218* rs11134527 A>G polymorphism was genotyped with TaqMan reagent. Allele-specific probes were purchased from Applied Biosystems. The PCR reaction was performed in 384-well plates that were run on an ABI-Q6 Sequence Detection System machine [24,25]. Moreover, to ensure the quality and accuracy of the genotyping results, we randomly selected 10% of the samples for repeat analysis, and the results were 100% concordant.

### Statistical analysis

First, we examined the Hardy–Weinberg equilibrium (HWE) of the samples. Next, the *χ*^2^ test was employed to assess the significant differences between cases and controls in the frequency distributions and genotypes. Odds ratios (ORs) and 95% confidence intervals (CIs) were used to quantify the association between the *miR-218* rs11134527 A>G polymorphism and Kawasaki disease susceptibility with adjustments for age and gender. The association between the *miR-218* rs11134527 A>G polymorphism and KD was assessed with analyses that were stratified by age, gender, and coronary artery lesions. The data analyses were performed with SAS software (Version 9.4; SAS Institute, Cary, NC, U.S.A.). A *P*-value <0.05 indicated a significant difference.

## Results

### Characteristics of the patients with Kawasaki disease

Table 1 presents the selected demographic characteristics of the cases and controls. There were no differences between the KD patients and controls in the distributions of age (*P*=0.602) or gender (*P*=0.143). The mean ages were 28.39 months for the KD patients (±24.68; range 1–166) and 28.48 months for the controls (±25.33; range 0.07–166). Among the cases, 31.39% and 68.61% were female and male, respectively, and the controls were 35.47% and 64.53% female and male, respectively (*P* 0.143). Among the cases, 31.58% had a CAL, and 61.42% had no coronary artery lesion (NCAL).

**Table 1:** Frequency distribution of selected variables for cases and controls

Variables	Cases (*n*=532)		Controls (*n*=623)		P^*^
	No.	%	No.	%	
Age range, month	1.00–166.0		0.07–166		0.602
Mean ± SD	28.39 ± 24.68		28.48 ± 25.33		
<12	137	25.75	165	26.48	
12–60	351	65.98	397	63.72	
>60	44	8.27	61	9.79	
Gender					0.143
Female	167	31.39	221	35.47	
Male	365	68.61	402	64.53	
Coronary artery lesion					
CAL	168	31.58			
NCAL	364	68.42			

Abbreviations: CAL, coronary artery lesion; NCAL, no coronary artery lesion.

* Two-sided *χ^2^* test for distributions between cases and controls.

### Association between the miR-218 rs11134527 A>G polymorphism and the risk of KD

The genotype distributions of the *miR-218* rs11134527 A>G polymorphism in the KD patients and controls are displayed in Table 2. The *miR-218* rs11134527 A>G genotype distribution analysis for HWE in the control group revealed an equilibrium (HWE = 0.779). However, there was no significant association between the *miR-218* rs11134527 A>G polymorphism and KD susceptibility after adjustments for age and gender (AG vs. AA: adjusted OR = 0.98, 95% CI = 0.75–1.27, *P*=0.863; GG vs. AA: adjusted OR = 1.13, 95% CI = 0.80–1.61, *P*=0.489; dominant model: adjusted OR = 1.01, 95% CI = 0.79–1.30, *P*=0.919; and recessive model: adjusted OR = 1.15, 95% CI = 0.84–1.57, *P*=0.380).

**Table 2:** Genotype distributions of rs11134527 A>G polymorphism and Kawasaki disease susceptibility

Genotype	Cases (*N*=523)	Controls (*N*=623)	P*	Crude OR (95% CI)	*P*	Adjusted OR (95% CI)^†^	*P*^†^
HWE = 0.779							
AA	162 (30.98)	195 (31.30)		1.00		1.00	
AG	267 (51.05)	328 (52.65)		1.00 (0.77–1.30)	1.000	0.98 (0.75–1.27)	0.863
GG	94 (17.97)	100 (16.05)		1.16 (0.81–1.64)	0.420	1.13 (0.80–1.61)	0.489
Additive			0.681	1.05 (0.89–1.25)	0.577	1.05 (0.88–1.25)	0.579
Dominant	361 (69.02)	428 (68.70)	0.906	1.02 (0.79–1.31)	0.906	1.01 (0.79–1.30)	0.919
Recessive	429 (82.03)	523 (83.95)	0.388	1.15 (0.84–1.56)	0.388	1.15 (0.84–1.57)	0.380

* χ^2^ test for genotype distributions between Kawasaki disease patients and controls.

† Adjusted for age and gender.

### Stratified analysis

Next, we further assessed the effects of the *miR-218* rs11134527 A>G polymorphism in the cases and controls with a stratified analysis (Table 3). The participants were stratified according to age, gender, and the presence of coronary artery lesions. In the analysis stratified by age, we found that there were more controls with the GG genotype among the children who were older than 60 months (adjusted OR = 0.26, 95% CI = 0.07–0.94, *P*=0.041).

**Table 3:** Stratification analysis for the association between rs11134527 A>G polymorphism and Kawasaki disease susceptibility

Variables	AA/AG	GG	Crude OR (95% CI)	*P*	Adjusted OR* (95% CI)	P*
	cases/controls					
Age, month						
<12	110/145	22/20	1.45 (0.75–2.79)	0.266	1.51 (0.78–2.94)	0.221
12–60	279/331	68/66	1.22 (0.84–1.78)	0.293	1.22 (0.84–1.78)	0.297
>60	40/47	4/14	0.34 (0.10–1.10)	**0.072**	**0.26 (0.07–0.94)**	**0.041**
Gender						
Females	130/189	35/32	1.59 (0.94–2.70)	0.086	1.56 (0.91–3.67)	0.104
Males	299/334	59/68	0.97 (0.66–1.42)	0.873	0.98 (0.67–1.43)	0.907
Coronary artery lesion						
CAL	140/523	26/100	0.97 (0.61–1.55)	0.904	0.97 (0.61–1.56)	0.912
NCAL	289/523	68/100	1.23 (0.88–1.73)	0.232	1.23 (0.87–1.73)	0.237

* Adjusted for age and gender.

## Discussion

In our study, the results did not reveal a significant relationship between the *miR-218* rs11134527 A>G polymorphism and KD susceptibility in Southern Chinese children. However, a stratified analysis suggested that there was a significant association between carriers of the AA/AG genotypes and the occurrence of KD compared with carriers of the GG genotype especially among the children older than 5 years.

KD is an acute systemic vasculitis and self-limiting disease that predominantly occurs in children younger than 5 years. The incidence rate of KD varies geographically, and it is more prevalent in Asian populations [26]. Although the etiology of KD remains unknown, extensively studies have suggested that the causes of KD may be affected by viral or bacterial infections, autoimmune factors and genetic factors [27]. With the goal of identifying the genetic factors related to KD, Kuo et al. [28] indicated that the C allele of *ITPKC* rs28493229 (341 KD patients and 1190 controls) is associated with the susceptibility to KD and aneurysm formation in KD patients in a Taiwanese population. A review revealed that many potential susceptibility genes are associated with the risk of KD and CAL, including SNPs of *ITPKC, CASP3, T helper type17, TGF-β, BLK, FCGR2A, KCNN2*, and other genes [29]. However, Natividad et al. [30] reported that they did not find any statistically significant associations of the *HLA-DRB1, TNF-α* and *ITPKC* genes with KD in KD patients relative to controls among a Filipino population. Other studies have reported similar results in that the susceptibilities to KD and CAL were not associated with the *HLA-DRB1* gene in a Taiwanese population and a Korean population [31,32]. These findings indicate that the results of such association studies may be affected by race, environment, and family genetics.

miRNAs are endogenous noncoding small RNAs that regulate the expression of genes by the reverse complementation of genes by specific miRNAs, and miRNAs are involved in the regulation of a variety of biological processes, including inflammation, cardiovascular diseases, and cancer. Polymorphisms in miRNAs gene may affect miRNA biogenesis and function; for example, the results from Tian et al. [33] suggested that the *miR-146a* rs2910164 and *miR-196a-2*rs11614913 polymorphisms were associated with the hepatitis virus-related hepatocellular cancer risk. Dai et al. [34] recruited 1143 subjects (583 controls; 560 breast cancer patients) and found that the T allele polymorphism of the miR-196a2 rs11614913 gene is associated with a decreased risk of breast cancer and that the *miR-499* rs3746444 AG/GG genotypes are associated with an increased risk of breast cancer in Chinese individuals. These authors also found that the *miR-196a-2*.rs11614913 and *miR-27a* rs895819 polymorphisms are correlated with reduced breast cancer risk [34,35]. Moreover, several studies have reported that miRNAs also play a critical role in KD. Researchers are focused on the potential of miRNAs as useful diagnostic biomarkers, therapeutic targets, and actors in disease pathogenesis in KD. Rong et al. [36] demonstrated that the serum *miR-92a-3p* levels were significantly higher in children with KD compared with non-Kawasaki disease subjects, and the *miR-92a-3p* level had a diagnostic sensitivity of 81.8% for KD. Zhang et al. [37] enrolled 102 patients with KD and 80 healthy controls in a study and found that the serum *miR-200c* and *miR-371-5p* levels were significantly higher in the KD patients compared with the controls. These two miRNAs were significantly higher in KD patients who were resistant to IVIG compared with those for whom IVIG was effective; thus, these two miRNAs may serve as diagnostic biomarkers and therapeutic targets in KD. In previous microRNAs target prediction studies, the results indicated that the target genes of serum *miR-200c, miR-371-5p*, and miR-145 are related to signaling pathways that include the *Wnt, MAPK, TGF-β*, and *mTOR* signaling pathways, and these pathways have been reported to be involved in inflammatory responses [38–40]. However, these findings provide no evidence that miR-218 plays an important role in KD.

*MiR-218* is a tumor suppressor gene that is expressed at significantly low levels in various tumors. Some targets of *miR-218*, such as *ROBO1, RICTOR, BIRC5* and *LAMB3*, have been reported to participate in many cancer signaling pathways, such as the *ERK/MAPK, Wnt/β-catenin*, and *Notch* pathways [12,41,42]. These findings indicate that the up-regulation of *miR-218* may reduce the risk of cancer by down-regulating these targets. SNPs of these miRNAs may improve miRNA binding affinity and alter the mRNA expression levels of the target genes and thus may contribute to the susceptibilities of humans to common diseases [43,44]. Therefore, we examined whether the *miR-218* rs11134527 A>G polymorphism is associated with the genetic susceptibility to KD. In our present study, we found no significant association between the *miR-218* rs11134527 A>G polymorphism and KD risk. Interestingly, we observed that the *miR-218* rs11134527 A>G carriers of the GG genotype were associated with a significantly decreased risk of KD compared with the AG/AA genotype carriers in the older-age subgroup (>60 months), which may be attributable to the fact that younger patients may have higher KD susceptibility, and the *miR-218* rs11134527 A>G variant homozygote GG genotype may affect *miR-218* binding and mRNA splicing, which would affect the expression of some targets. Zhou et al. [45] demonstrated that the *miR-218* rs11134527 GG genotype is associated with an increased risk of cervical cancer compared with the AA genotype among Chinese women. Furthermore, according to a previous studies of cardiovascular disease, Gao et al. [46] and Chen et al. [47] reported no associations of *miR-218* rs11134527 A>G with the risks of congenital heart disease or myocardial infarction in a Chinese population. To some extent, these studies may support our results.

Although this is the first investigation of the association between the *miR-218* rs1113452 A>G polymorphism and KD risk in Chinese children, our study has potential limitations that should be reviewed. First, due to the retrospective nature of the original study design, we did not have detailed information about other factors, such as medical histories, parental environmental exposures, and dietary intakes. Second, we only performed a case–control study to explore the association between the *miR-218* rs1113452 A>G polymorphism and KD susceptibility, and we did not explore the expression level of miR-218 in the peripheral blood or the potential mechanisms of action of the polymorphism. Third, we only examined the rs1113452 A>G polymorphism and other potential SNPs of *miR-218* were not included the present study.

In short, we recruited 1155 children (532 cases and 623 controls) to participate in our research. Compared with previous studies (samples of <120 cases), this project had a relatively larger sample size. Moreover, this is the first report to demonstrate that the *miR-218* rs11134527 A>G polymorphism was not associated with the genetic susceptibility to KD; however, the *miR-218* rs11134527 A>G polymorphism may have a weak effect on KD risk among those older than 5 years in the Chinese population. Further investigation of the mechanisms by which the *miR-218* rs11134527 A>G polymorphism affects KD susceptibility are required.

## Abbreviations

CAL: coronary artery lesion
CI: confidence interval
HWE: Hardy–Weinberg equilibrium
IVIG: intravenous immunoglobulin
KD: Kawasaki disease
miRNA: microRNA
NCAL: no coronary artery lesion
OR: odds ratio
SNP: single nucleotide polymorphism
